# Efficacy and Synergy of Small Molecule Inhibitors Targeting *FLT3*-ITD^+^ Acute Myeloid Leukemia

**DOI:** 10.3390/cancers13246181

**Published:** 2021-12-08

**Authors:** Javier Bregante, Anna Schönbichler, Daniel Pölöske, Lina Degenfeld-Schonburg, Garazi Monzó Contreras, Emir Hadzijusufovic, Elvin D. de Araujo, Peter Valent, Richard Moriggl, Anna Orlova

**Affiliations:** 1 Institute of Animal Breeding and Genetics, University of Veterinary Medicine, 1210 Vienna, Austria; bregante@mpi-cbg.de (J.B.); Anna.Schoenbichler@vetmeduni.ac.at (A.S.); Daniel.Poeloeske@vetmeduni.ac.at (D.P.); garazi.monzo@gmail.com (G.M.C.); richard.moriggl@vetmeduni.ac.at (R.M.); 2 Department of Medicine I, Division of Hematology and Hemostaseology, Medical University of Vienna, 1090 Vienna, Austria; lina.degenfeld-schonburg@meduniwien.ac.at (L.D.-S.); emir.hadzijusufovic@meduniwien.ac.at (E.H.); peter.valent@meduniwien.ac.at (P.V.); 3 Ludwig Boltzmann Institute for Hematology and Oncology, Medical University of Vienna, 1090 Vienna, Austria; 4 Clinic for Companion Animals and Horses, University Clinic for Small Animals, Internal Medicine Small Animals, University of Veterinary Medicine, 1210 Vienna, Austria; 5 Department of Chemical and Physical Sciences, University of Toronto Mississauga, Mississauga, ON L5L1C6, Canada; e.dearaujo@mail.utoronto.ca; 6 Centre for Medicinal Chemistry, University of Toronto Mississauga, Mississauga, ON L5L1C6, Canada

**Keywords:** acute myeloid leukemia, tyrosine kinase inhibitor, FMS-like tyrosine kinase 3, targeted therapy, ponatinib, cabozantinib, WS6, ispinesib

## Abstract

**Simple Summary:**

*FLT3*-ITD mutations belong to the most frequent yet most detrimental genetic alterations in AML. Next-generation FLT3 inhibitors are potent therapeutics and often effective in AML patients carrying the FLT3-ITD driver kinase. However, AML cells are particularly quick in acquiring resistance to FLT3 kinase blockers. We aimed to identify novel therapeutic options for *FLT3*-ITD^+^ AML, to investigate possible emerging resistance mechanisms to FLT3 inhibitors and to explore alternative targeting strategies. We applied a kinase-focused drug screen to find alternative therapeutics. We identified ispinesib, a kinesin spindle blocker, and kinase blockers WS6, ponatinib and cabozantinib, as very efficacious agents against *FLT3*-ITD^+^ AML cells. Importantly, we identify the combination of cabozantinib and ispinesib as particularly potent against *FLT3*-ITD^+^ AML. We suggest that a combinatorial treatment with these drugs could overcome resistance mechanisms and kill *FLT3*-ITD^+^ AML blasts.

**Abstract:**

Constitutive activation of FLT3 by ITD mutations is one of the most common genetic aberrations in AML, present in ~1/3 of cases. Patients harboring *FLT3*-ITD display worse clinical outcomes. The integration and advancement of FLT3 TKI in AML treatment provided significant therapeutic improvement. However, due to the emergence of resistance mechanisms, *FLT3*-ITD^+^ AML remains a clinical challenge. We performed an unbiased drug screen to identify 18 compounds as particularly efficacious against *FLT3*-ITD^+^ AML. Among these, we characterized two investigational compounds, WS6 and ispinesib, and two approved drugs, ponatinib and cabozantinib, in depth. We found that WS6, although not yet investigated in oncology, shows a similar mechanism and potency as ponatinib and cabozantinib. Interestingly, ispinesib and cabozantinib prevent activation of AXL, a key driver and mechanism of drug resistance in *FLT3*-ITD^+^ AML patients. We further investigated synergies between the selected compounds and found that combination treatment with ispinesib and cabozantinib or ponatinib shows high synergy in *FLT3*-ITD^+^ AML cell lines and patient samples. Together, we suggest WS6, ispinesib, ponatinib and cabozantinib as novel options for targeting *FLT3*-ITD^+^ AML. Whether combinatorial tyrosine kinase and kinesin spindle blockade is effective in eradicating neoplastic (stem) cells in *FLT3*-ITD^+^ AML remains to be determined in clinical trials.

## 1. Introduction

Acute myeloid leukemia (AML) is a hematopoietic malignancy defined by an abnormal proliferation and accumulation of poorly differentiated myeloid progenitor cells [1]. AML constitutes the most common acute leukemia in adults, but despite innovative therapeutic strategies, largely based on targeted tyrosine kinase inhibitors (TKI) or chemotherapeutic intervention, ~70% of patients >65 of age still succumb to the disease within one year after diagnosis [2,3]. A reason for this poor prognosis is genetic heterogeneity, clonal evolution of cancer cells and recurrent mutations that lead to vulnerable tyrosine kinase signaling cascades, which characterize AML [1]. Altogether, this leads to the lack of novel targeted therapeutic for these patients.

*FLT3*-ITD (FMS-like tyrosine kinase 3-internal tandem duplication) is a frequent gain-of-function genetic alteration, reported in 25–35% of AML cases [3,4]. FLT3 serves as a growth factor receptor displaying intrinsic kinase activity and it is normally expressed by bone marrow stromal cells where it promotes cell survival and proliferation via signaling pathways such as phosphatidylinositol-3 kinase (PI3K)-AKT-mTOR activation, RAS-RAF-MAPK cascade triggering and the signal transducer and activator of transcription 5 (STAT5) engagement [5,6,7]. *FLT3*-ITD^+^ AML cells display hyperactivation of a number of kinase pathways, including tyrosine kinases such as AXL, KIT, SRC-family, ERBB, JAK and FLT3 kinases and also serine/threonine kinases, such as PIM, CDK4/6, or CDK7/8/9. Mutated FLT3 contains replicated sequences in its juxtamembrane (JM) domain, which constitutively activate FLT3 kinase [3]. These sequences vary between patients in location and length and can determine the severity of the disease outcome. Longer ITD regions are, for example, particularly associated with a worse disease outcome, as they can increase, among other mechanisms, downstream STAT5 activation [8]. Overall, the *FLT3*-ITD alteration has an established unfavorable effect on patient prognosis and is particularly associated with high leukemic burden, poor overall survival and risk of relapse after conventional chemotherapy [9,10].

Inhibitors that target FLT3 in *FLT3*-ITD^+^ AML are often divided into first-generation and next-generation inhibitors [11]. First-generation inhibitors, such as midostaurin, target a broad spectrum of kinases, associated with higher off-target effects. Additionally, they show limited single-agent activity in relapsed *FLT3*-ITD^+^ AML [11,12]. In contrast, next-generation FLT3 inhibitors, such as gilteritinib and quizartinib, demonstrated high single-agent potency, confirming that targeting FLT3 is indeed a promising approach [13,14,15]. However, even these potent inhibitors are not able to control the disease in all cases and resistance-conferring point mutations were observed in patients receiving these agents [16]. In some cases, different FLT3 kinase inhibitors could even generate non-overlapping resistance mutations in a single FLT3 receptor molecule [17]. An additional observed resistance mechanism of AML cells to FLT3 inhibitors also includes the activation of alternative tyrosine kinase signaling pathways, such as the activation of the tyrosine kinase receptor AXL and other only partly investigated pathways in AML [18,19,20]. Overall, FLT3 was proven to be an achievable and effective target. Importantly, the rapid emergence of resistance to FLT3 inhibitors emphasizes the need to investigate and identify new specific compounds. 

An alternative strategy to avoid and overcome resistance, which is increasingly applied in the treatment of many cancer types, is to combine different agents that target and inhibit different signaling pathways and may thus work synergistically [21]. These combinatorial treatments additionally allow treatment with lower drug dosages, decreasing off-target and side-effects, often improving both patient compliance and outcomes and can account for the heterogeneity between cancers [22].

In this study, we present an unbiased evaluation of 679 approved and investigational compounds. We identify that the combination of cabozantinib and ispinesib acts synergistically and potently against *FLT3*-ITD^+^ AML. Validation was performed in cell lines as well as in primary patient samples. Conclusively, we contribute to *FLT3*-ITD^+^ AML research by evaluating new TKI compounds for future treatment and by supporting the concept that combinatorial treatments are a solution to enhance drug efficacy, preventing potential resistance mechanisms.

## 2. Results

### 2.1. A High-Throughput Drug Screen Identifies Compounds That Exhibit a Strong Inhibitory Activity against FLT3-ITD^+^ AML

The development of potent next-generation FLT3 inhibitors has provided improvements in the therapeutic outcome for AML patients harboring *FLT3*-ITD mutations. However, particularly due to the rapid emergence of resistance, the overall and progression-free survival of *FLT3*-ITD^+^ AML patients remains poor [10,16].

**Figure 1:**
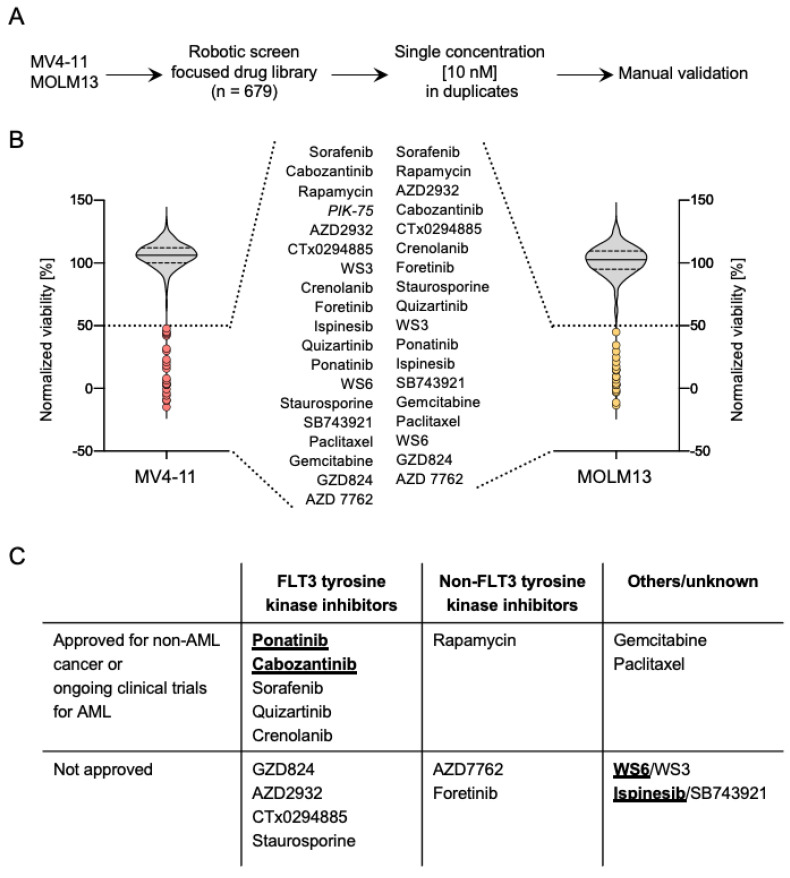
A high-throughput screen using a 679 small molecule inhibitor library. (**A**) Illustration of the applied screening procedure. Cell viability was measured after 72 h of treatment using CellTiter-Glo^®^ (Promega). (**B**) Violin plots showing the effect of the drugs on the viability of MV4-11 and MOLM13. Drugs that reduced the viability below 50% are depicted in red (MV4-11) or yellow (MOLM13) and are listed according to their effect from lowest (top) to the highest (bottom). Drug (PIK-75) that induced decreased viability only in MV4-11 shown in italics. (**C**) Discovered hits separated according to their clinical use status and the known mechanism of action. Drugs investigated further shown in underlined bold.

Aiming to identify vulnerable nodes in *FLT3*-ITD^+^ AML, so as to increase the therapeutic armamentarium for this disease, we performed high-throughput drug screening and evaluated the effect of a cancer drug library at a single dose (10 nM) on the viability of two *FLT3*-ITD^+^ AML cell lines, MV4-11 and MOLM13 (Figure 1A). The screening was performed in duplicates for each cell line and showed a high level of reproducibility between replicates (Table S1). Similarly, the drug screening yielded a high correlation between the two cell lines, suggesting that we obtained a robust data set to explore the mechanism of drug action (r = 0.85, *p* < 0.0001) (Figure S1A). Collectively, these results indicate that the drug screening carried out in two AML cell lines yielded a dataset of particularly potent small molecule inhibitors against AML.

Top hits from the drug screen were defined as compounds that showed a reduction in viability below 50% in both cell lines, as seen in Figure 1B. Remarkably, we found that of the 19 different drugs that yielded a viability below 50% in MV4-11 cells, 18 were shared between both cell lines (Figure S1B). A summary of the hits from the screen, including the type of inhibitor and its clinical stage in development, is detailed in Figure 1C.

We decided to follow up on the characterization of ponatinib and cabozantinib, two approved FLT3 TKI, which are currently enrolled in clinical trials for repurpose in *FLT3*-ITD^+^ AML. We further included two non-approved drugs, namely WS6 and ispinesib, in our characterization. Ponatinib is a TKI targeting the T315I point mutation of BCR-ABL that is currently approved for chronic myeloid leukemia (CML) and Ph^+^ acute lymphoblastic leukemia (ALL) [23]. Cabozantinib is a multi-targeted TKI that is currently approved for renal cell carcinoma and hepatocellular carcinoma [24]. Both ponatinib and cabozantinib were reported to target FLT3. Cabozantinib additionally inhibits AXL and VEGFR2 [24]. Interestingly, both ponatinib and cabozantinib were included in the BEAT AML study, where both acted more efficacious in *FLT3*-ITD^+^ samples compared to *FLT3*-ITD^−^ (Figure S1E) [25]. Ispinesib, as well as its analog SB743921, are cytoskeleton disruptors that target the kinesin spindle protein, which is required for cell division [26]. Ispinesib was previously tested in advanced solid cancers. Interestingly, WS6 and co-discovered progenitor WS3, were initially identified as promoters of β-cell proliferation [27]. Erb3 binding protein (EBP)-1 and the IκB kinase (IKK) pathway were reported as the mechanism of WS3/6 action [27]. An overview of the effect on cell viability of the hits identified in the screening is shown in Figure S1C and a comparison of the chemical structure of the compounds chosen for further characterization is depicted in Figure S1D.

### 2.2. WS6, Ponatinib and Cabozantinib Are Selective for FLT3-ITD^+^ Compared to FLT3-wt AML

**Figure 2:**
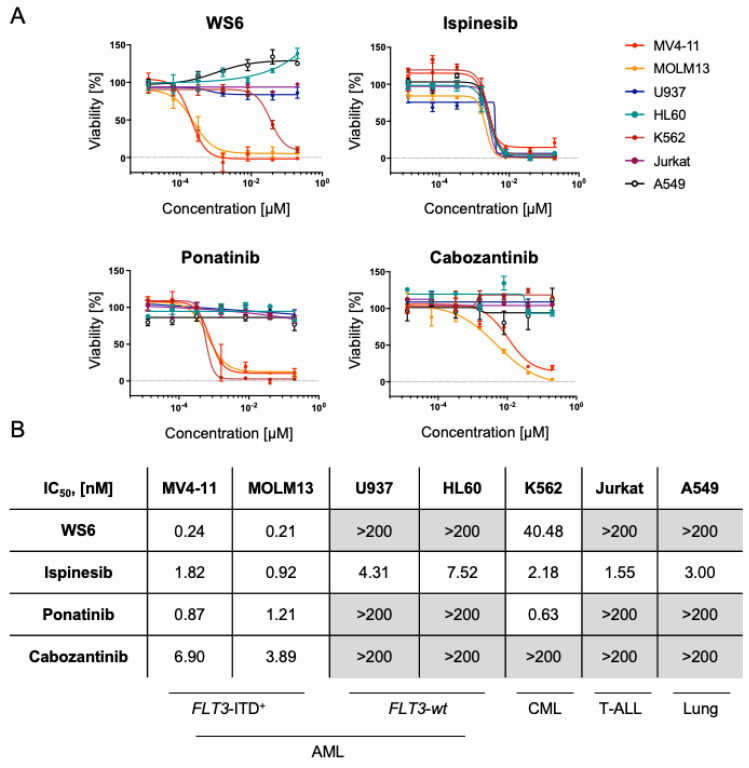
Selectivity of chosen compounds in various AML, CML and negative control cancer cell lines. (**A**) Cytotoxicity assays of leukemia cell lines treated with the indicated compounds for 72 h. Each cell line was treated with the compound of interest and the viability was determined using CellTiter-Blue^®^ (Promega). Jurkat and A459 cancer cell lines serve as negative controls. Representative dose-response curves are shown, error bars represent mean ± SEM, *n* = 3. (**B**) Average IC_50_ values of cell lines treated with WS6, ispinesib, cabozantinib, or ponatinib. Values above 200 nM are depicted in grey.

To evaluate the selectivity of the chosen compounds, we evaluated the activity of the drugs in *FLT3*-ITD^+^ AML (MV4-11 and MOLM13), *FLT3*-*wt* AML cell lines (U937 and HL60), other hematopoietic cancer cell lines, including CML (K562) and two negative control cancer cell lines, namely T-ALL (Jurkat), as well as a lung adenocarcinoma cell line (A549). *FLT3*-ITD^+^ AML lines were extremely sensitive to ponatinib, cabozantinib and WS6 (IC_50_ < 4 nM). Respectively, these compounds displayed limited activity targeting *FLT3*-*wt* cell lines (IC_50_ > 200 nM). As expected, ponatinib also targeted the BCR-ABL^+^ K562 cell line (IC_50_ = 0.63 nM). Surprisingly, WS6 also affected K562 at 40.48 nM (Figure 2A,B). Expectedly, ispinesib showed broad-spectrum antineoplastic activity and displayed high activity, targeting all the screened tumor cell lines at 3 nM IC_50_ on average (Figure 2A,B).

Additionally, we sought to compare the in vitro efficacy of these compounds in *FLT3*-ITD^+^ AML cell lines with the current standard of care compounds used for *FLT3*-ITD^+^ malignancies, including the broad spectrum chemotherapeutic cytarabine and the FDA-approved FLT3 inhibitors midostaurin and gilteritinib. Strikingly, the screened compounds WS6, ispinesib, ponatinib and cabozantinib displayed a lower IC_50_ than the FDA-approved compounds for *FLT3*-ITD^+^ AML (Figure S2A,B). We additionally evaluated the effect of WS6 and ispinesib on healthy bone marrow stem cells. We observed no significant effect of WS6 or ispinesib at 1 µM on CD34^+^/CD38^−^/CD45^dim^ Annexin V levels, indicating a reasonable therapeutic window for both compounds (Figure S2C,D).

### 2.3. WS6, Ponatinib and Cabozantinib Inhibit the FLT3-STAT5 Axis 

**Figure 3:**
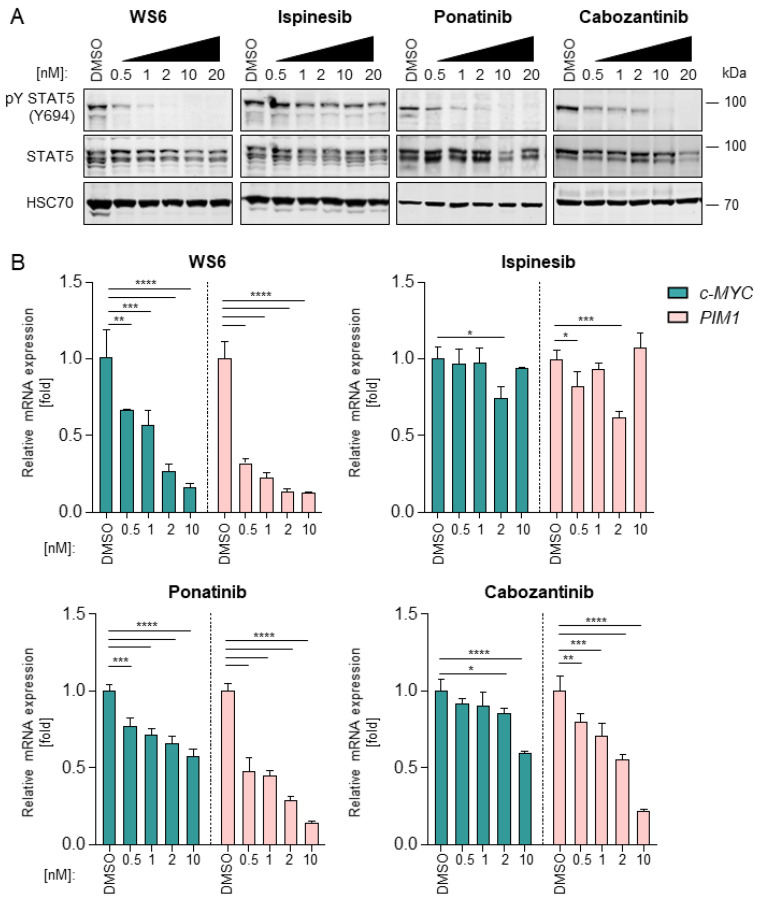
Impact of four selected compounds on the FLT3-STAT5 pathway. (**A**) MV4-11 cells were treated with WS6, ispinesib, cabozantinib, or ponatinib at the indicated concentrations for 24 h. Thereafter, the expression of activated STAT5 (pY STAT5), STAT5 and heat shock cognate 70 (HSC70), used as loading control, was analyzed via Western blotting. Representative experiment shown, *n* = 2. Uncropped images can be found in Figures S6 and S7. (**B**) MOLM13 cells were treated with WS6, ispinesib, cabozantinib or ponatinib at the indicated concentrations for 24 h. Thereafter, *c-MYC* and *PIM-1* gene expression levels were analyzed via RT-qPCR. Cq values were normalized to the housekeeping gene *GAPDH*. Representative experiment shown, *n* = 2. * *p* < 0.05, ** *p* < 0.01, *** *p* < 0.001, **** *p* < 0.0001.

The *FLT3*-ITD mutation leads to a constitutive ligand-independent activation of downstream signaling pathways, including STAT5 and subsequent *MYC* and *PIM* expression [28]. Given that previous investigations described a downregulating effect of cabozantinib and ponatinib on the STAT5-MYC interplay [29], we decided to investigate this aspect for WS6 and ispinesib. We confirmed that the tyrosine phosphorylation of STAT5 was effectively inhibited by cabozantinib and ponatinib in a dose-response manner in both MV4-11 and MOLM13 cells. As expected, the total STAT5 levels remained unchanged upon treatment (Figure 3A and Figure S3A). Ispinesib did not significantly affect pY STAT5 levels, validating that its mechanism of action is independent of oncogenic STAT5 signaling. Surprisingly, WS6 affected STAT5 activation by blocking its tyrosine phosphorylation, similarly to ponatinib and cabozantinib.

Next, we sought to evaluate the effect of selected compounds on STAT5 target genes. In particular, the influence of FLT3 inhibitors on *c-MYC* and *PIM-1* expression was assessed. It was found that *c-MYC* expression was downregulated by WS6, ponatinib and cabozantinib (Figure 3B and Figure S3B). Similarly, *PIM-1* expression was decreased by WS6, ponatinib and cabozantinib in a dose-dependent manner. Remarkably, WS6 induced the most potent inhibition of *c-MYC* and *PIM-1* compared to cabozantinib and ponatinib. Ispinesib did not efficiently block *c-MYC* or *PIM-1* expression, validating that its mode of action is independent of the FLT3-STAT5 axis. Thus, ispinesib targets a different core cancer pathway excluding STAT5 oncogene signaling, suggesting it might be more suitable for combinatorial drug targeting [30].

### 2.4. Ispinesib and WS6 Induce Apoptosis More Rapidly Than Ponatinib and Cabozantinib

**Figure 4:**
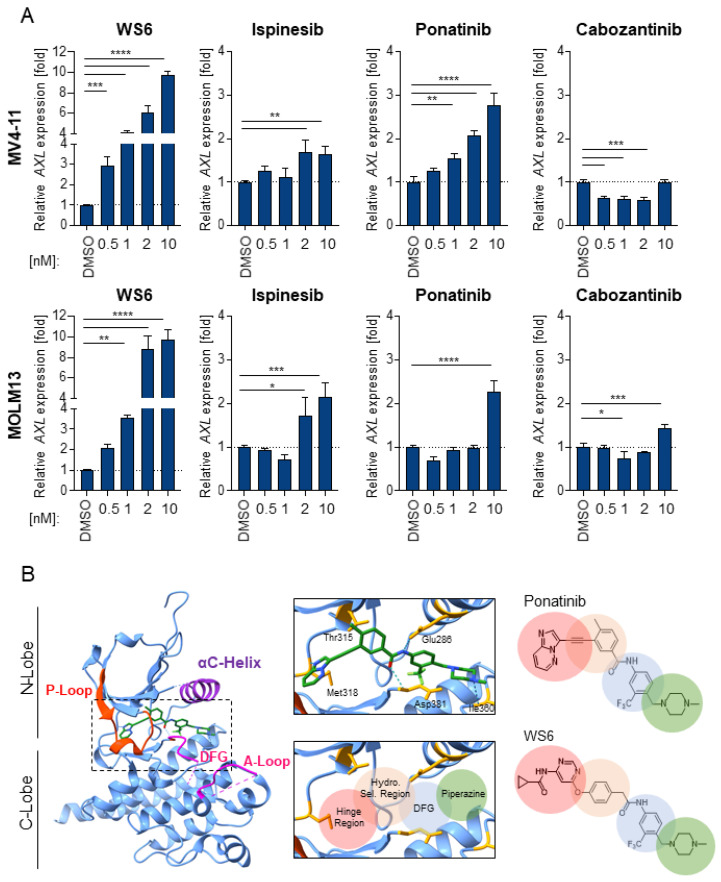
Insights into resistance and apoptosis mechanisms of screened compounds, followed by structural comparison of WS6 and ponatinib. (**A**) MV4-11 and MOLM13 cells were treated with WS6, ispinesib, cabozantinib, or ponatinib at the indicated concentrations for 24 h. Thereafter, *AXL* gene expression levels were analyzed via RT-qPCR. Cq values were normalized to the housekeeping gene *GAPDH.* A representative experiment is shown from *n* = 2. * *p* < 0.05, ** *p* < 0.01, *** *p* < 0.001, **** *p* < 0.0001. (**B**) Structure of ponatinib (green) bound to the kinase domain of BCR-ABL with key structural motifs labelled (PDB:3OXZ). The inset illustrates a subset of residues responsible for critical binding interactions and H-bonds are shown in blue dotted lines. The shaded circles indicate the regions of interaction that are likely preserved between ponatinib and WS6. Figures were generated with Chimera [31].

To expand our understanding of the molecular mechanisms and efficacy of FLT3 inhibitors we explored the effects of compound treatment on the tyrosine kinase receptor AXL. It was shown that AXL overexpression, mediated mainly by an enhanced and prolonged pY STAT5 activation, constitutes a key mechanism of escape from FLT3 inhibitors [19,20]. Here, we investigated how selected compounds affect *AXL* mRNA levels in MV4-11 and MOLM13 using RT-qPCR. We observed that WS6 dramatically increased *AXL* expression. Ponatinib and ispinesib also upregulated *AXL* expression, albeit at higher concentrations starting at 1 or 2 nM, respectively. Cabozantinib downregulated *AXL* up to a concentration of 10 nM, which is in line with its known inhibitory activities (Figure 4A). This constitutes a promising approach to bypass secondary relapse due to the emergence of *FLT3*-ITD^+^ AML-resistant clones because of AXL axis upregulation.

To further study the molecular mechanisms of the identified compounds, we investigated the effect of WS6, ispinesib, ponatinib and cabozantinib treatment on the apoptosis pathways of cancer cells. Both MV4-11 and MOLM13 showed a dose-dependent increase of Caspase 3/7 activity upon treatment with all used inhibitors. Interestingly, WS6 and ispinesib were most potent and significantly increased Caspase 3/7 activity displaying augmented activity already at 2 nM concentration. Notably, ponatinib and cabozantinib had less impact on treated AML cells and cabozantinib could induce a significant increase in Caspase 3/7 activity only at high concentrations (>50 nM) (Figure S4C).

Annexin V/propidium iodide (PI) staining confirmed the observed trend. Here, cells were treated for 24 h with the compounds, stained with both agents and subsequently analyzed via flow cytometry. We observed a significant increase in Annexin V single-stained and Annexin V/PI double-stained cells after 5 nM WS6 and ispinesib treatment in both cell lines compared to the DMSO control. Ponatinib and cabozantinib, on the other hand, enabled less Annexin V and PI to bind at the same concentration after 24 h treatment (Figure S4A,B). We also validated these findings by observing a dose-response dependent increase of cleaved PARP upon inhibitor treatment (Figure S5A, Uncropped western blots, please view Figure S8).

### 2.5. WS6 Action Shows Similarities to Ponatinib and Cabozantinib

Due to the similarities between the efficacy and mechanism of small molecule inhibitor WS6 and two well-established FLT3 inhibitors, cabozantinib and ponatinib that we compared here, coupled with the structural similarity to ponatinib, we hypothesized that WS6 possesses tyrosine kinase inhibition activity. To investigate this, first, we utilized the SwissTargetPredition tool to predict putative targets of WS6 [32]. Indeed, results show that the most probable targets of WS6 are tyrosine kinases, including ABL1, EGFR, as well as SRC family kinases (Table 1 and Table S2).

Ponatinib is a type II kinase inhibitor and third-generation small molecule drug targeting BCR-ABL. The core structure exploits several key features of known kinase inhibitors to target the (i) hinge region, (ii) hydrophobic selectivity pocket and (iii) DFG-out pocket of BCR-ABL (Figure 4B) [33]. The hinge region consists of a linker peptide that connects the two core kinase domains (N-lobe and C-lobe) and engages in multiple H-bonds and van der Waal interactions with the adenine ring of ATP. The hydrophobic selectivity pocket is deeper behind the ATP-binding site. The DFG-out pocket is revealed within an inactive kinase state and corresponds to a conformation with a critical Mg^2+^-coordinating aspartate residue pointing away from the ATP-binding site. This DFG-out pocket is a distinctive binding site occupied by type II kinase inhibitors.

The fused aromatic rings (imidazole [1,2-b] pyridazine) of ponatinib directly participate in H-bond interactions with the hinge region and the linker ethynyl moiety allows the methylphenyl ring to access the hydrophobic selectivity pocket behind the ATP-binding site. The tri-fluoromethyl-phenyl ring engages with the C-Lobe and DFG-out pocket of the kinase. The methylpiperazine participates in H-bonding with the kinase backbone, as well as van der Waal’s interactions [34].

Notably, congruent structural elements are also observed in WS6, suggesting a similar binding mode as ponatinib (Figure 4B). WS6 preserves the tri-fluoromethyl-phenyl ring and solubilization moiety methylpiperazine, likely retaining the interactions highlighted above. The pyrimidine and adjacent amide-linked cyclopropyl offer comparable H-bonding partners for hinge region binders, as seen in similar monocyclic derivatives of ponatinib [35]. The critical difference is the ether linkage, in place of the ethynyl, which likely alters the phenyl ring positioning within the hydrophobic selectivity pocket, as well as interactions with the gate-keeper residue to this pocket. In ponatinib, the linear ethynyl can sterically evade the native or mutated gate-keeper residue (Thr315 in BCR-ABL), while the ether in WS6 may allow for hydrogen-bonding interactions similar to first-generation inhibitors, such as imatinib.

### 2.6. Ispinesib Synergizes with Cabozantinib and Ponatinib in Inhibiting AML Cells Growth

**Figure 5:**
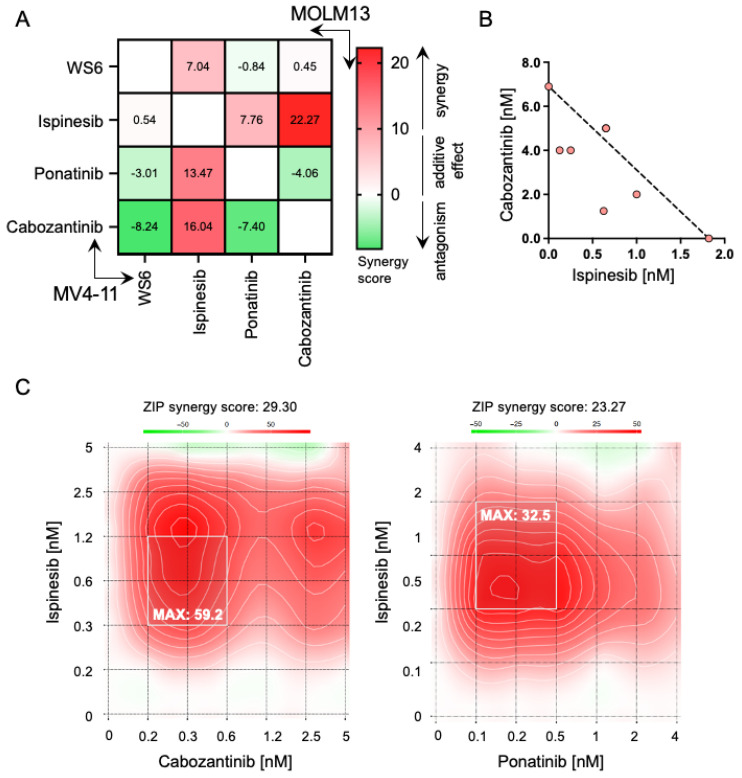
Interaction landscape of compounds with non-overlapping targets reveals an improved targeting impact. (**A**) Heatmap summarizing average ZIP scores of the synergy experiments between WS6, ispinesib, cabozantinib and ponatinib in MV4-11 and MOLM13, *n* = 3. (**B**) Isobologram of the ispinesib and cabozantinib interaction in MV4-11 cell line. (**C**) Representative interaction landscape between ponatinib or cabozantinib and ispinesib in MV4-11. The most synergistic area is shaded and the maximum synergy score is indicated. ZIP stands for Zero interaction potency, *n* = 3.

The emergence of resistance mechanisms has been considered one of the major limitations of the application of FLT3 inhibitors in clinics [16]. Consequently, we support the hypothesis that the use of combinatorial therapies might prevent the emergence of FLT3 inhibitor-related resistances [21]. The complete and sustained depletion of *FLT3*-ITD has been highlighted as essential for the successful elimination of the malignant clone [36], hence we initially investigated the synergistic effect between two FLT3 inhibitors identified in the screening, ponatinib and cabozantinib. To analyze the efficacy of drug combinations, the web application SynergyFinder was used [37,38]. Interestingly, and in line with this concept, we found that the combination of the utilized FLT3 inhibitors was antagonistic rather than synergistic (Figure 5A). The same trend was observed when combining both, ponatinib and cabozantinib with WS6. Remarkably, a similar interaction landscape was found in both *FLT3*-ITD^+^ cell lines.

Alternatively, we evaluated the combination of the cytoskeleton disruptor, ispinesib and FLT3 inhibitors. This combination yielded a strong synergistic effect on both MV4-11 and MOLM13 (Figure 5B,C). Particularly, the combination of ispinesib with cabozantinib was strongly synergistic (Figure 5B). This suggests that the combination of compounds that present different targets in separated core cancer pathways, such as spindle assembly or STAT5 oncogenic signaling, is beneficial to achieve a synergistic effect. To validate this finding, we studied the combination of ispinesib with current standard of care drugs for *FLT3*-ITD^+^ AML, including broad-spectrum chemotherapeutic drugs (cytarabine), approved FLT3 inhibitors (midostaurin, gilteritinib and quizartinib) and the BCL2 family member blocker venetoclax. We observed that combining broad-spectrum compounds (cytarabine and ispinesib) yielded an antagonistic effect, which is in line with our previous observation of combining compounds that share targets. On the other hand, the combination of ispinesib with FLT3 inhibitors and venetoclax achieved an additive effect, containing clear areas of synergies that can be further explored (Figure S5B,C). Overall, this demonstrates that the combination of inhibitors that target overlapping pathways possesses a limited added therapeutic effect, but quite separate drug target combinations could pave new therapeutic avenues.

### 2.7. Selected Compounds as Well as Cabozantinib-Ispinesib Combination Are Effective in Leukemic Cells Derived from FLT3-ITD^+^ AML Patients

**Figure 6:**
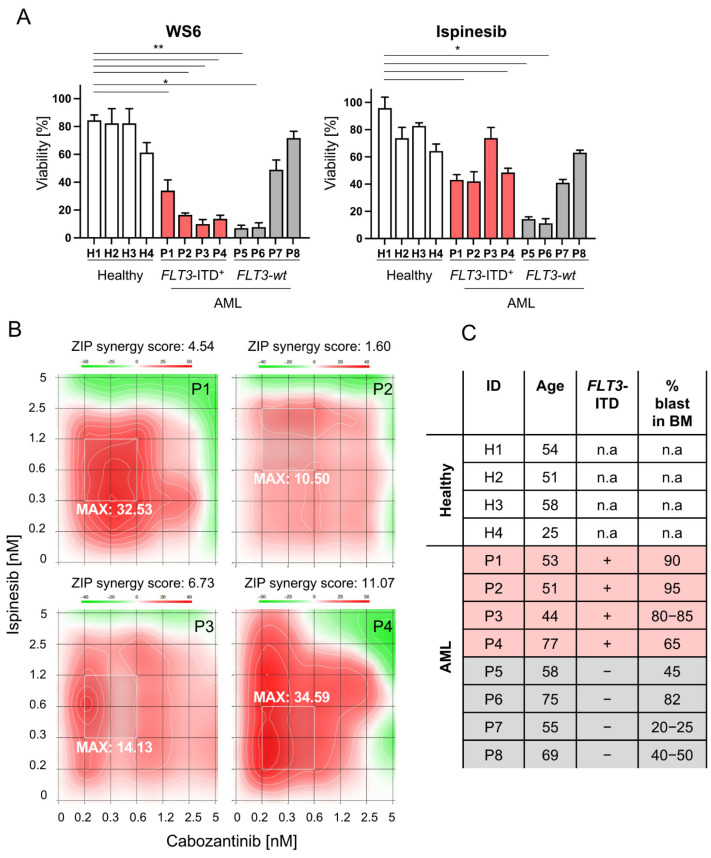
Combination of ispinesib and cabozantinib in *FLT3*-ITD^+^ AML patient samples displays marked drug synergy. (**A**) Effect of 1 µM WS6 or ispinesib on viability of patient bone marrow samples and healthy controls. Viability was measured after 48 h using CellTiter-Glo^®^ and normalized to DMSO control. * *p* < 0.05, ** *p* < 0.01. (**B**) Representative interaction landscape between cabozantinib and ispinesib in bone marrow samples from *FLT3*-ITD^+^ patients. The most synergistic area is shaded and maximum synergy score is indicated. ZIP stands for Zero interaction potency. (**C**) Overview on the cytogenetics, age and percentage of blast in bone marrow (BM) of involved patients.

Finally, we compared the efficacy of the non-approved compounds WS6 and ispinesib in bone marrow samples of healthy controls and AML patients, among which four harbored a *FLT3*-ITD rearrangement and four expressed wildtype *FLT3*. We observed that WS6 had a significant effect on the viability of *FLT3*-ITD^+^ samples, while displaying no effect on healthy samples. Interestingly, this compound also had a significant effect on two out of four *FLT3*-*wt* patient samples, which highlights their heterogeneity. Furthermore, analysis of the WS6 targeting profile will be needed to evaluate its effect on *FLT3-wt* AML. Ispinesib had no effect on healthy cells, but displayed a significant effect on both *FLT3*-ITD^+^ and *FLT3*-*wt* samples, which is in accordance with the above results in AML cell lines (Figure 6A). This highlights the potential of using both WS6 and ispinesib for AML therapy due to their therapeutic window. We also observed that some patient bone marrow cells (P4, P5, P7) died to a greater extent than the percentage of the blasts present in the bone marrow. We conclude that the portion of the non-blast bone marrow population is also affected and its impact needs to be investigated further.

Next, the synergy between ispinesib and cabozantinib was validated in patient samples. We observed that the combination of these two compounds yielded an additive effect in four bone marrow samples derived from *FLT3*-ITD^+^, with a ZIP synergy score ranging from 1.60 to 11.07. Interestingly, the most synergistic area overlaps for all the patients (Figure 6B). An overview of patient samples and cytogenetics is shown in Figure 6C. Overall, this confirms the promise to include ispinesib in the therapeutic armamentarium for AML. Our drug combination exercise also reveals that non-overlapping or further separated cancer pathway targets can limit the emergence of resistance that limits patients’ lifespans. Further work is needed to explore combinatorial targeting and unwanted toxicity in the direction of improved targeted therapies for AML.

## 3. Discussion

Despite recent advances in the treatment of AML, the long-term survival rate is unacceptably low and new therapeutic strategies are urgently needed for patients, particularly those who cannot undergo transplantation and exhibit high-risk molecular profiles regarding relapse, such as the *FLT3*-ITD^+^ subset of AML. Identification of novel selective FLT3 inhibitors, but also innovative and combinatorial treatment strategies are urgently needed to reduce the emergence of drug resistances and to improve the status quo of AML treatment.

Using high-throughput drug screening, we provide an unbiased comparison of approved and investigational drugs that are translatable for *FLT3*-ITD^+^ AML treatment. The selected compounds WS6, ispinesib, ponatinib and cabozantinib were further validated through a combination of cytotoxicity assays, gene expression studies and synergy analyses, both in cell lines and patient samples. We suggest WS6 as a broad-range tyrosine kinase inhibitor that might see further exploration in AML and potentially other cancer treatments. Additionally, our data show novel applications of the spindle-inhibitor ispinesib and we found an efficacious combination between ispinesib and cabozantinib, which could be a valuable option for patients with resistant *FLT3*-ITD^+^ AML, failing to respond to targeted FLT3 inhibitors.

The small molecule inhibitor WS6, as well as its analog WS3, were remarkably active in the drug screen, despite the absence of previous identification of activity of these compounds in AML or any other cancers. The compounds were initially identified in a screening for factors that promote β-cell proliferation [27]. WS3 and WS6 primarily target EBP1, a member of the DNA/RNA-binding protein family ERB1-4 that is implicated in the regulation of cell growth, apoptosis and differentiation [39,40]. Apart from this, they also modulate the IκB kinase, which is part of the NFκB pathway [27]. Here, a comparison of chemical structures and mechanisms of action between WS6 and the TKI ponatinib led us to conclude that WS3/WS6 exhibit previously unknown TKI qualities, capable of targeting FLT3 and ABL1 kinases. Briefly, WS6 compound contains structural elements, such as the tri-fluoromethyl-phenyl ring, methylpiperazine and comparable H-bonding partners for hinge region binders, likely acting similarly to ponatinib. In addition, WS6 was also potent in the downregulation of STAT5 target genes and in inducing apoptosis in *FLT3*-ITD^+^ AML cells. Based on this, WS6 may be a new small-molecule candidate for clinical trials in *FLT3*-ITD^+^ AML. However, further studies would be required to investigate a reliable targeting profile and the potential clinical development of WS6.

Ispinesib and its analog SB743921 inhibit the kinesin spindle protein, which is a motor protein required for the adequate formation of the bipolar mitotic spindle [26]. Ponatinib and cabozantinib are two chemically distinct classes of TKI. Both drugs target FLT3, are approved for treatment of different types of cancer and are currently also being investigated in clinical trials for treating AML. Ispinesib, on the other hand, has been investigated in solid cancer clinical trials but did not show sufficient efficacy [41]. In our study, ispinesib was very effective in killing *FLT3*-ITD^+^ cells, albeit not targeting the same pathway as the other drugs in the screen. Off-target toxicity evaluations revealed that ispinesib had no cytotoxic effect on healthy stem cells, which is expected as it targets rather fast-dividing cells. The TKIs in the study were more selective in targeting only *FLT3*-ITD^+^ cells. The study of off-target toxicity profiles is, next to the evaluation of IC_50_ values, an important step for treatment translation to the clinics. When drugs show minimal off-target toxicity, evaluated IC_50_ doses might be exceeded in treatment and if off-target toxicity is high, IC_50_ doses might lose their relevance. The present results indicate that WS6 and ispinesib might be used in higher doses than revealed in the potency studies in patients and they might support a lower dose of TKI in a combinational treatment, without resulting in side effects.

Systems biology insights revealed that combinatorial treatments of cancer cells can be more effective when divergent pathways are targeted [30]. Different angles of targeting might also account for the genetic heterogeneity of *FLT3*-ITD^+^ AML and divergently mutated leukemic clones in patients. In previous studies around *FLT3*-ITD^+^ AML combinatorial treatments, FLT3 inhibitors were combined with bromodomain and extra-terminal domain (BET) protein inhibitors and DNA methyltransferase (DNMT) inhibitors [42,43]. These approaches were generally promising and acknowledging this, we combined low doses of ispinesib with the other investigated drugs in this study. Indeed, ispinesib demonstrated high synergy with cabozantinib and ponatinib, while these drugs did not synergize with each other. We propose that the limited selectivity of ispinesib in patients could thus be significantly enhanced when it is combined with a targeted drug. Moreover, targeted tyrosine kinase inhibition could be successfully supported by the inhibition of mitotic spindle of rapidly dividing AML cells by ispinesib. On the contrary to ponatinib, WS6 did not synergize with ispinesib, which might hint that WS6 targets other main pathways in the cell.

The *FLT3*-ITD^+^ AML subtype is specifically characterized by rapid mutations and the upregulation of various tyrosine kinases cascades [3]. FLT3-tyrosine kinase domain (TKD) mutations are associated with primary resistance to FLT3 inhibitors and are detected in 7–11% of patients with AML, occurring mainly at positions D835 or I836 [44,45]. Interestingly, other FLT3-TKI resistance mechanisms include upregulation of *AXL* mRNA, AXL tyrosine kinase activation levels, upregulation of BCL-XL, overexpression of RAD51 and Cyclin D3 mutations, which altogether illuminate escape routes via STAT5 activation [18,19,20,46,47,48]. Our data also indicate that STAT5 targeting might be beneficial, since further FLT3 inhibitor escape routes like FLT3-TKD, BCL-2 family member upregulation, enhanced cell cycle progression through D-type cyclin, c-Myc and CDK4/6 action are also under STAT5 control in AML [44,45]. Furthermore, acquired gain-of-function mutations in JAK1, JAK2, or JAK3 and oncogenic RAS mutations were discovered as further FLT3-TKI-resistance mechanisms [49].

The treatment of *FLT3*-ITD^+^ AML patients is complicated by the rapid emergence of resistance against treatment. We therefore investigated whether the selected compounds triggered the previously described STAT5-AXL escape pathway in their mechanism of action. AXL, a member of the TAM receptor family, is strongly activated in many different cancer types and was shown to drive resistance of AML cells to FLT3 inhibitors via signals from the stromal microenvironment and the activation of STAT5. Interestingly, WS6 and ponatinib indeed switched their signaling to AXL, which might lead to fast resistance development. In contrast, cabozantinib, that also targets AXL, had a minimal inhibitory effect on *AXL* expression when used in moderate doses. This is in line with the blocking of pY STAT5 and suggests its applicability in relapsed patients resistant to first-line FLT3 inhibitors. High concentrations of ispinesib and notably also cabozantinib, interestingly increased *AXL* mRNA expression, which calls for a further explanation and suggests that AXL can also be upregulated by other kinds of cellular stress, such as mitotic disruption [19]. We therefore suggest that cabozantinib is an option for relapsed patients who are resistant to first-line FLT3 inhibitors. As discussed above, cabozantinib might be even more potent in combination with ispinesib.

In conclusion, we provided a rationale for more potent combinatorial AML therapies exploring targeting of distinct core cancer pathways. We identified WS6 and ispinesib as very promising compounds and we could validate the high efficacy of ponatinib and cabozantinib in AML treatment. We shed light on potential new targets of WS6 as a highly effective TKI, previously described as a predominantly EBP1 modulator. We show that its action profile largely mimics ponatinib and the target prediction and chemical structure comparison support this finding. We investigated synergies between the best four compounds identified by drug screening and standard of care treatment options and discovered that the combination of TKI with a kinesin spindle protein inhibitor, specifically cabozantinib/ponatinib with ispinesib, is highly synergetic. Combining drugs that target non-overlapping pathways might be the best way to enhance the action of single drugs to achieve high synergy, leading to less toxicity and thereby improving the lives of patients.

## 4. Materials and Methods

### 4.1. Cell Lines and Compounds

All cell lines were purchased from DSMZ (Braunschweig, Germany) or ATCC (Manassas, VA, USA). The cell lines were regularly tested to exclude mycoplasma contamination and authenticated. All cell lines were grown at 37 °C and 5% CO_2_. A549 cells were cultured in DMEM medium (Gibco™, Thermo Fisher Scientific, Waltham, MA, USA). All other cell lines were cultured in RPMI 1640 medium (Gibco™, Thermo Fisher Scientific). Both media were supplemented with 10% fetal bovine serum (FBS), 10 U/mL penicillin, 10 µg/mL streptomycin and 2 mM L-glutamine (all Gibco™, Thermo Fisher Scientific). Bortezomib (S1013; Selleck Chemicals, Houston, TX, USA), cabozantinib (S1119; Selleck Chemicals), cytarabine (PHR1787; Millipore Sigma, Burlington, MA, USA), gilteritinib (HY-12432; MedChemExpress, Monmouth Junction, NJ, USA), ispinesib (HY-50759; MedChemExpress), midostaurin (M1323; Millipore Sigma), ponatinib (11494; Cayman Chemical, Ann Arbor, MI, USA), quizartinib (17986; Cayman Chemical), venetoclax (HY-15531; MedChemExpress) and WS6 (S7442; Selleck Chemicals) were dissolved in dimethyl sulfoxide (DMSO; Carl Roth, Karlsruhe, Germany) and diluted further in culture medium immediately before use.

### 4.2. Patient Samples

BM cells (iliac crest) of patients with AML were collected at diagnosis and stored in a local biobank until used. The study was approved by the ethics committee of the Medical University of Vienna (1184/2014 and 1334/2021) and conducted in accordance with the declaration of Helsinki. All patients gave written informed consent. Diagnoses were established according to French-American-British (FAB) and World Health Organization (WHO) criteria. Cells were maintained in RPMI 1640 supplemented with 10% FBS, 10 U/mL penicillin, 10 µg/mL streptomycin, 2 mM L-glutamine. Viability of the samples was normalized to the vehicle (DMSO) control.

### 4.3. High-Throughput Compound Screening

For the high-throughput drug screening, a compound library that contained 679 approved and investigational compounds was used. The screening was carried out in duplicates in two AML *FLT3*-ITD^+^ cell lines, MV4-11 and MOLM13. The compounds were initially transferred into 384-well plates using an Echo^®^ acoustic liquid handler (Labcyte, San Jose, CA, USA). 1000 cells per well in 50 nL DMSO (Carl Roth) were seeded on top of the drugs using a dispenser (Thermo Fisher Scientific) to achieve a total volume of 50 µL/well. Cells were incubated for 72 h and cell viability was measured using a CellTiter-Glo^®^ Luminiscent Cell Viability Assay (Promega, Madison, WI, USA) according to the manufacturer’s instructions in an EnVision^®^ multimode plate reader (PerkinElmer, Waltham, MA, USA). All drugs were tested at a single 10 nM dose. Cell viability was determined by normalizing values to a negative and a positive control as percentages, using linear regression individually for each plate. DMSO (Carl Roth) treated cells were used as a negative control and values were set to 100% survival in the data analysis. Bortezomib-treated cells were used as a positive control and values were set to 0% survival in the data analysis.

### 4.4. Cytotoxicity Assay

To determine the half-maximal inhibitory concentration (IC_50_) of the selected compounds on various cell lines, CellTiter-Blue^®^ cell viability assays or CellTiter-Glo^®^ cell viability assays (both Promega) were performed. For this, cells were seeded in 96-well flat bottom plates at a cell density of 10,000 cells/well. Cells were treated in triplicates with the compound of interest at various concentrations or with 10 μM Bortezomib as a positive control. Cell viability of treated cell lines was measured using CellTiter-Blue^®^ after 72 h incubation, whereas cell viability of treated patient samples was measured using CellTiter-Glo^®^ after 48 h incubation. Plates were measured using a GloMax^®^ plate reader (Promega) and IC_50_ values were determined by non-linear regression.

### 4.5. Synergy Screening

To measure synergy, cells were seeded in 96-well flat bottom plates at a cell density of 10,000 cells/well. Cells were treated with various combinations of the compounds of interest (matrix-like), with single compound dilution series or 10 μM Bortezomib as a positive control. Cell viability of treated cell lines was measured using CellTiter-Blue^®^ after 72 h. The drug combination evaluation was done using SynergyFinder [37] or isobolograms. The reference model used to quantify the synergism between compounds was the Zero interaction potency (ZIP) model.

### 4.6. Immunoblotting

Sample preparation and Western blotting were performed using standard techniques. Nitrocellulose membranes (0.45 µm Amersham Protran; 10600002; GE Healthcare, Buckinghamshire, UK) were incubated with the following antibodies at the dilution indicated: monoclonal rabbit anti-phospho-STAT5 (Y694) antibody (9351; 1:1000; Cell Signaling Technologies, Danvers, MA, USA), monoclonal mouse anti-STAT5 antibody (610191; 1:1000; BD Biosciences Pharmingen, San Jose, CA, USA), polyclonal rabbit anti-PARP antibody (9542; 1:1000; Cell Signaling Technologies), monoclonal mouse anti-HSC70 antibody (SC-7298, 1:5000, Santa Cruz, St. Louis, MO, USA).

### 4.7. Gene Expression Analysis

RNA isolation was performed using an RNeasy Mini Kit (Qiagen, Hilden, Germany) according to the manufacturer’s instructions. RNA reverse transcription was carried out using the RevertAid First Strand cDNA synthesis kit with oligo(dT)18 primers (Thermo Fisher Scientific, Waltham, MA, USA). The reaction program included 60 min at 42 °C, 10 min at 72 °C and a final cooling step at 4 °C. The primers used for quantitative PCR (qPCR) are shown in Table 2. Samples were prepared by combining forward and reverse primers (10 µM each), GoTaq^®^ qPCR Master Mix (Promega) and 12.5 ng/µL of cDNA, diluted in H_2_O. The qPCR reaction was carried out using a Bio-Rad CFX96 Touch Real-Time System (Bio-Rad Laboratories, Hercules, CA, USA) according to the protocol: 95 °C 2 min; 40× (95 °C 15 s; 60 °C 1 min); 95 °C 1 min. The comparative Cq method (2^−ΔΔCq^ method) was used for the analysis of RT-qPCR data and *GAPDH* was used as the housekeeping gene. Each experiment was done in triplicates.

### 4.8. CD34^+^ Annexin V/DAPI Staining

To determine drug-induced apoptosis mononuclear cells (MNC) of three lymphoma patients without bone marrow (BM) infiltration, cells were incubated in control medium (untreated) or in medium supplemented with different compounds at 37 °C for 48 h. Then, cells were washed and stained with APC-H7-conjugated monoclonal antibody (mAb) against CD45, PE-conjugated mAb against CD34, APC-conjugated mAb against CD38 and FITC-conjugated mAb against Annexin V. 4′,6-Diamidin-2-phenylindol (DAPI) was added before apoptosis was analyzed by multi-color flow cytometry on a FACS Canto (BD Biosciences, San José, CA, USA). Stem cells were gated as CD34^+^/CD38^−^/CD45^dim^ cells and apoptosis was quantified measuring Annexin V positive cells using FlowJo software (Version: 10.7.1, BD Biosciences).

### 4.9. Annexin V/PI Staining

To quantify (percentage) apoptotic cells upon compound treatment, an Annexin V-FITC/PI apoptosis detection kit (BD Biosciences Pharmingen) was used according to the manufacturer’s instructions. Briefly, MV4-11 and MOLM13 cells were seeded in 6-well culture plates at a density of 1 × 10^6^ cells per well and treated with WS6, ispinesib, ponatinib (2 nM, 5 nM) and cabozantinib (10 nM, 20 nM) for 72 h. DMSO and bortezomib (2 μM) treated cells were used as controls. Cells were washed twice in PBS, resuspended in Annexin Binding Buffer and stained with the Annexin V-FITC and PI (all BD Biosciences). After 15 min of incubation, cells were analyzed on a FACSCanto (BD Biosciences). Analysis was done with the FlowJo Software (Version: 10.7.1; BD Biosciences). Cells were gated in four quadrants in which double-negative cells accounted for live cells, Annexin V single-positive cells accounted for cells in early apoptosis, Annexin V and PI double-positive cells accounted for cells in late apoptosis and PI single-positive cells constituted necrotic cells and cell debris.

### 4.10. Caspase 3/7 Activity Assay for Apoptosis Detection

To investigate Caspase 3/7 activity upon compound treatment, a Caspase-Glo^®^ 3/7 Assay System (Promega) was used according to the manufacturer’s instructions. Briefly, MV4-11 and MOLM13 cells were seeded in 96-well flat-bottom plates at a cell density of 10,000 cells per well. Cells were treated in triplicate with WS6, ispinesib, ponatinib (2 nM, 5 nM and 10 nM) and cabozantinib (10 nM, 20 nM and 50 nM) for 72 h. DMSO was used as a control. After adding Caspase-Glo^®^ 3/7 reagent (Promega) to the cells (1:1 ratio) and 2 h of incubation, Caspase activity was measured using a GloMax^®^ plate reader (Promega). Normalization was done by dividing the Caspase 3/7 activity values by the values of a CellTiter-Blue^®^ (Promega) viability assay with the same set-up. Values are shown as fold change to the DMSO control values. Each experiment was done in triplicate.

### 4.11. Statistical Analysis

Statistical analysis was performed using the GraphPad Prism 9.1.1 (GraphPad Software, Inc., San Diego, CA, USA) and the data are reported as mean values ± SD, unless otherwise stated. In the case of multiple comparison, a one-way ANOVA test with Bonferroni correction was applied. Multiple comparisons were calculated by comparing each group to the control group. A *p*-value below 0.05 was considered statistically significant (* *p* < 0.05, ** *p* < 0.01, *** *p* < 0.001, **** *p* < 0.0001).

## 5. Conclusions

In summary, we validated WS6, ispinesib, ponatinib and cabozantinib as efficacious drugs against AML. We showed that the combination of ispinesib and cabozantinib can be beneficial in resistant AML. Hereby, we provide alternative strategies for improved AML treatment approaches for further validation in clinical trials.

## Figures and Tables

**Table 1:** Top 10 predicted WS6 targets according to the SwissTargetPrediction tool. Full list can be found in Table S2.

**Target**	**Protein Name**	**Target Class**
MAP kinase signal-integrating kinase 2	MKNK2	Kinase
MAP kinase-interacting serine/threonine-protein kinase MNK1	MKNK1	Kinase
Tyrosine-protein kinase SRC	SRC	Kinase
Serine/threonine-protein kinase B-RAF	BRAF	Kinase
Tyrosine-protein kinase ABL	ABL1	Kinase
Tyrosine-protein kinase LCK	LCK	Kinase
Vascular endothelial growth factor receptor 2	VEGFR-2/KDR	Kinase
Tyrosine-protein kinase TIE-2	TEK	Kinase
Tyrosine-protein kinase Lyn	LYN	Kinase
Serine/threonine-protein kinase RAF	RAF1	Kinase

**Table 2:** Primers used for quantitative real-time PCR.

Gene	Sequence	Fragment Size
*c-MYC*	fwd: TTTCGGGTAGTGGAAAACCA	90 bp
	rev: CACCGAGTCGTAGTCGAGGT	
*PIM-1*	fwd: CTCAAGCTCATCGACTTCGG	105 bp
	rev: ATGGTAGCGGATCCACTCTG	
*AXL*	fwd: GTTTGGAGCTGTGATGGAAGGC	120 bp
	rev: CGCTTCACTCAGGAAATCCTCC	
*GAPDH*	fwd: AAGGTGAAGGTCGGAGTCAA	108 bp
	rev: AATGAAGGGGTCATTGATGG	

## Data Availability

The data presented in this study are available on request from the corresponding author.

## Supplementary Materials

The following are available online at https://www.mdpi.com/article/10.3390/cancers13246181/s1, Figure S1. Drug screen reveals novel potent compounds, Figure S2. Identified compounds are more efficacious than standard of care treatment, Figure S3. Impact of the selected compounds on the FLT3-STAT5 pathway, Figure S4. Selected compounds induce apoptosis, Figure S5. Synergy assessment between ispinesib and standard of care drugs, Figure S6. Uncropped western blots for Figure 3A and S3A, Figure S7. Uncropped western blots for Figure 3A and S3A, Figure S8. Uncropped western blots for Figure S5A. Table S1: Drug Screen. Table S2: SwissTargetPrediction.
