# Supplementary material for: Efficacy and Synergy of Small Molecule Inhibitors Targeting FLT3-ITD+ Acute Myeloid Leukemia
Source: Cancers (Basel). 2021 Dec 8;13(24):6181. doi: 10.3390/cancers13246181 (PMC8699584; doi:10.3390/cancers13246181)
Supplement: Supplementary file 1 [file cancers-13-06181-s001.zip › cancers-1494412-Figures S1-S8.pdf]

cancers

MDPI

Supplementary Materials

Efficacy and Synergy of Small Molecule Inhibitors Targeting *FLT3*-ITD⁺ Acute Myeloid Leukemia

Javier Bregante, Anna Schönbichler, Daniel Pölöske, Lina Degenfeld-Schonburg, Garazi Monzó Contreras, Emir Hadzijusufovic, Elvin D. de Araujo, Peter Valent, Richard Moriggl and Anna Orlova

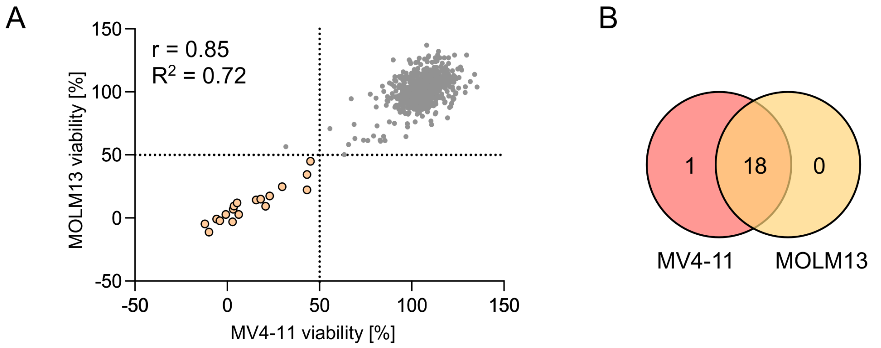

C

	Viability [%]	
	MV4-11	MOLM13
AZD 7762	-9.83	-11.24
GZD824	-12.03	-4.72
Gemcitabine	-5.76	-0.99
Paclitaxel	-3.89	-2.33
WS6	2.98	-3.16
SB743921	-0.70	2.68
Ispinesib	6.27	2.83
Ponatinib	3.31	6.99
Quizartinib	3.71	9.37
Staurosporine	5.41	11.88
Foretinib	15.76	14.33
WS3	20.79	9.33
Crenolanib	18.16	14.87
CTx0294885	23.03	17.41
AZD2932	29.78	24.77
Cabozantinib	43.25	22.28
Rapamycin	43.26	34.35
Sorafenib	46.49	44.98

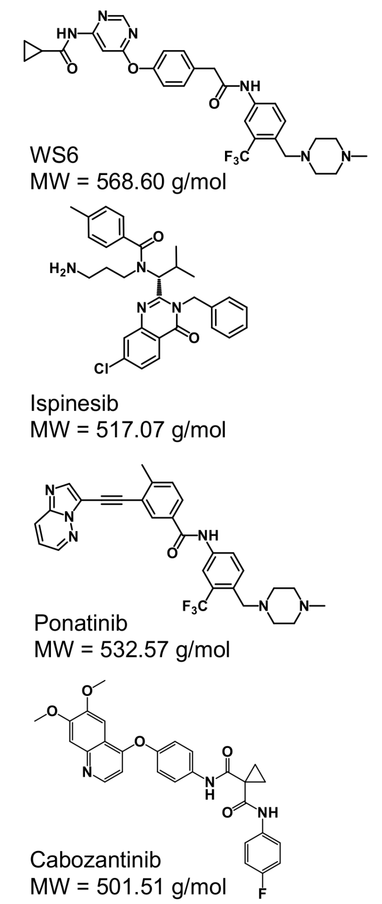

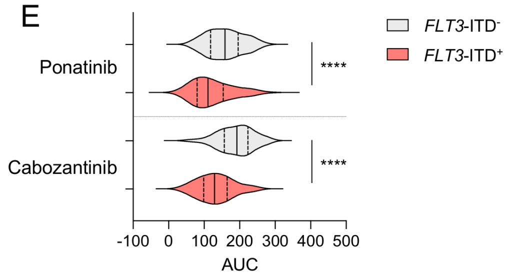

Supplementary Figure S1. Drug screen reveals novel potent compounds. (A) Correlation of the screening results in MV4-11 and MOLM13. Compounds that reduce viability of both cell lines below 50% depicted in yellow. **(B)** Venn diagram showing the overlap of the hits in the two cell lines, MV4-11 and MOLM13. **(C)** List of 18 compounds and the viability from the high-throughput screen, compounds investigated further marked with yellow. **(D)** Structures and molecular weight of selected compounds. **(E)** Effect of ponatinib and cabozantinib on *FLT3*-ITD⁺ and *FLT3*-ITD⁻ samples in BEAT AML dataset.

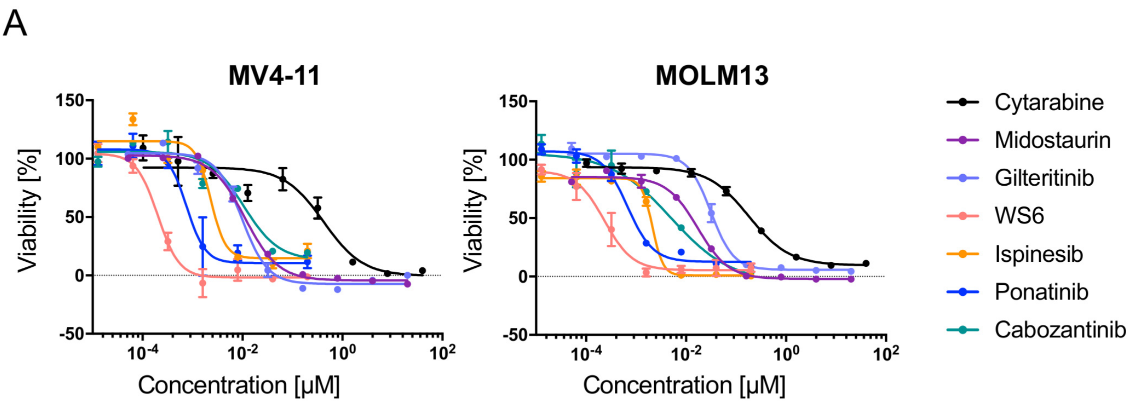

B

IC$_{50}$, [nM]	WS6	Ispinesib	Ponatinib	Cabozan.	Cytarabine	Midost.	Gilter.
**MV4-11**	0.24	1.82	0.87	6.90	418.40	11.19	9.73
**MOLM13**	0.21	0.92	1.21	3.89	180.10	17.08	5.16

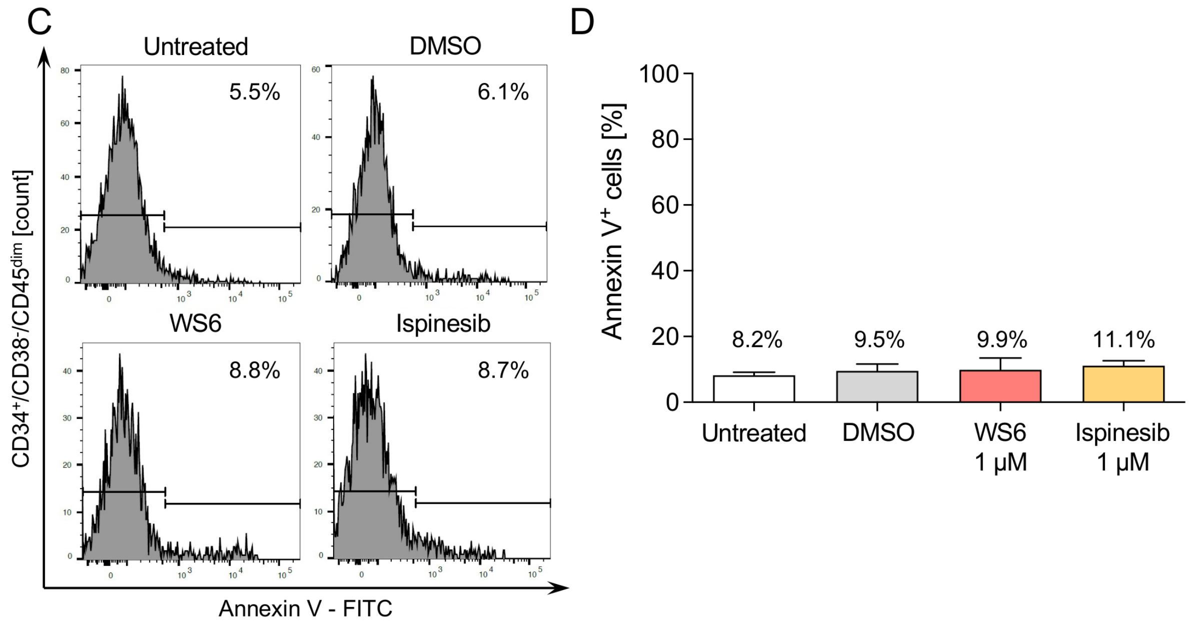

Supplementary Figure S2. Identified compounds are more efficacious than standard of care treatment. (A) Cytotoxicity assay of MV4-11 cells and MOLM13 cells treated with indicated compounds for 72 h. Viability was determined with Cell Titer-Blue® Cell Viability Assay. Representative dose-response curves are shown, error bars represent mean +/- SEM, n=3. **(B)** IC$_{50}$ values of treated cell lines. **(C)** Representative Annexin V staining of CD34$^+$/CD38$^-$/CD45^{dim} bone marrow cells after treatment with compounds for 48 h. **(D)** Quantification of Annexin V staining of CD34$^+$/CD38$^-$/CD45^{dim} bone marrow cells after treatment with compounds or 48 h, error bars represent mean +/- SEM , n=3. The numbers above the bars indicate average.

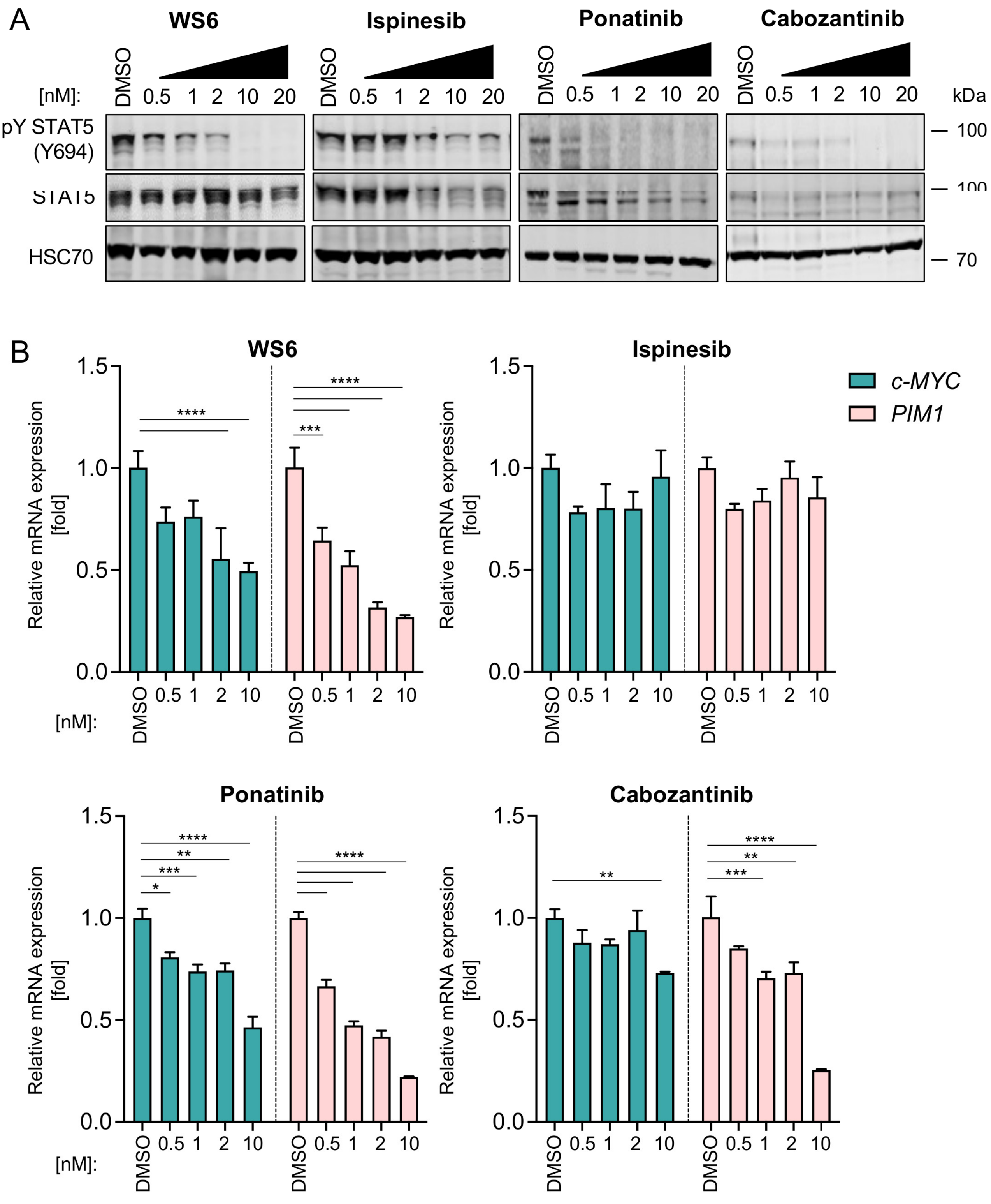

Supplementary Figure S3. Impact of the selected compounds on the FLT3-STAT5 pathway. (A) MOLM13 cells were treated with WS6, ispinesib, cabozantinib or ponatinib at the indicated concentrations for 24 h. HSC70 was used as loading control, n=2. Uncropped images can be found in **FigureS6** and **Figure S7**. **(B)** MV4-11 *c-MYC* and *PIM-1* gene expression at different concentrations of WS6, ispinesib, cabozantinib or ponatinib. Cells were treated with compounds at the indicated concentrations for 24 h. Cq values were normalized to the housekeeping gene *GAPDH*, n=2.

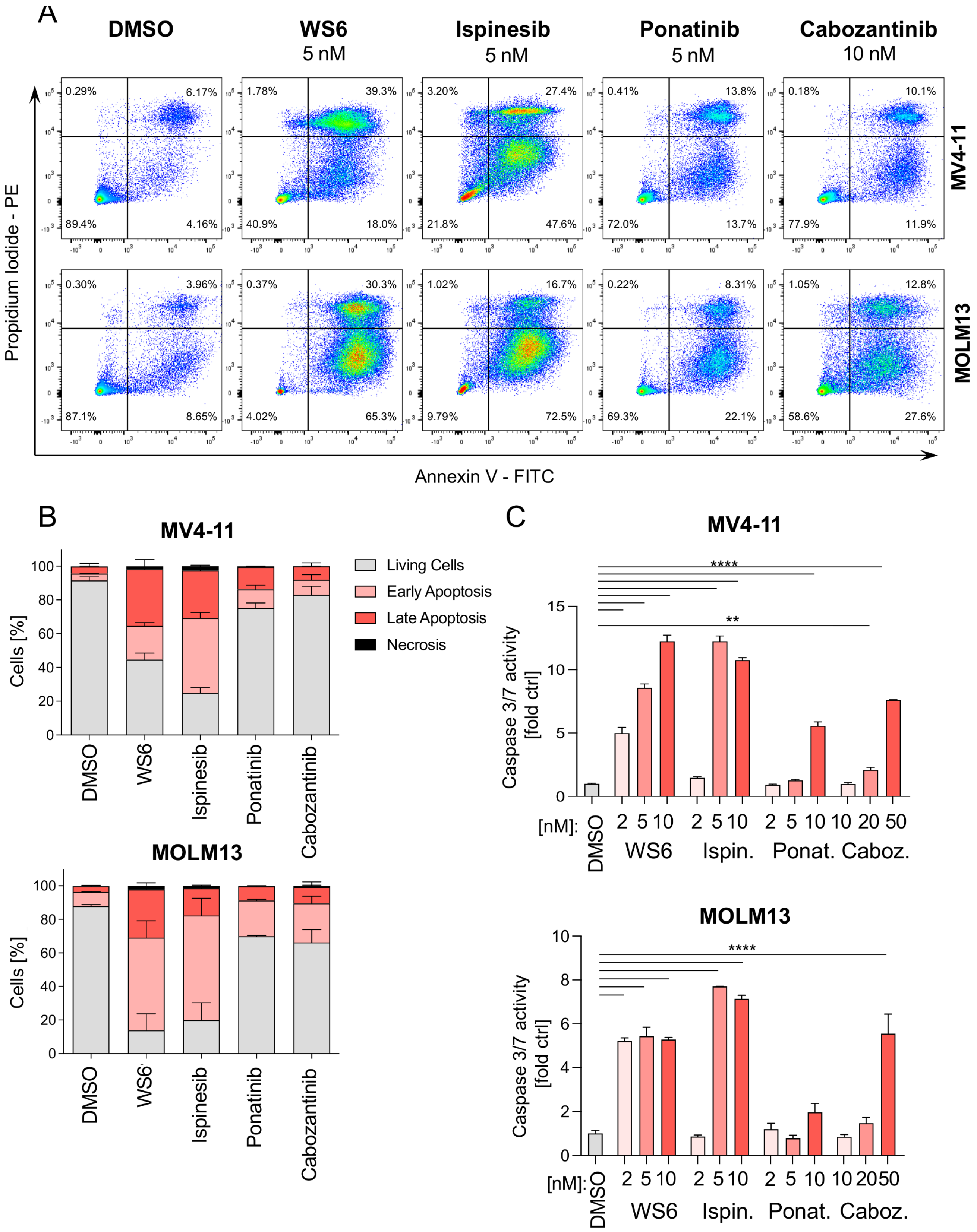

Supplementary Figure S4. Selected compounds induce apoptosis. (A) Representative flow cytometry plots using Annexin V/PI staining for apoptosis. MV4-11 and MOLM13 cells were treated for 24 h with compounds at indicated concentrations and subsequently stained with Annexin V-FITC/PI-PE, n=2. **(B)** Quantification of Annexin V/PI staining results. **(C)** MV4-11 and MOLM13 cells were treated with WS6, ispinesib, cabozantinib or ponatinib at the indicated concentrations for 24 h. Thereafter, Caspase 3/7 activity was measured using Caspase-Glo® 3/7 reagent (Promega). Values are shown as fold change to the DMSO control values, n=2.

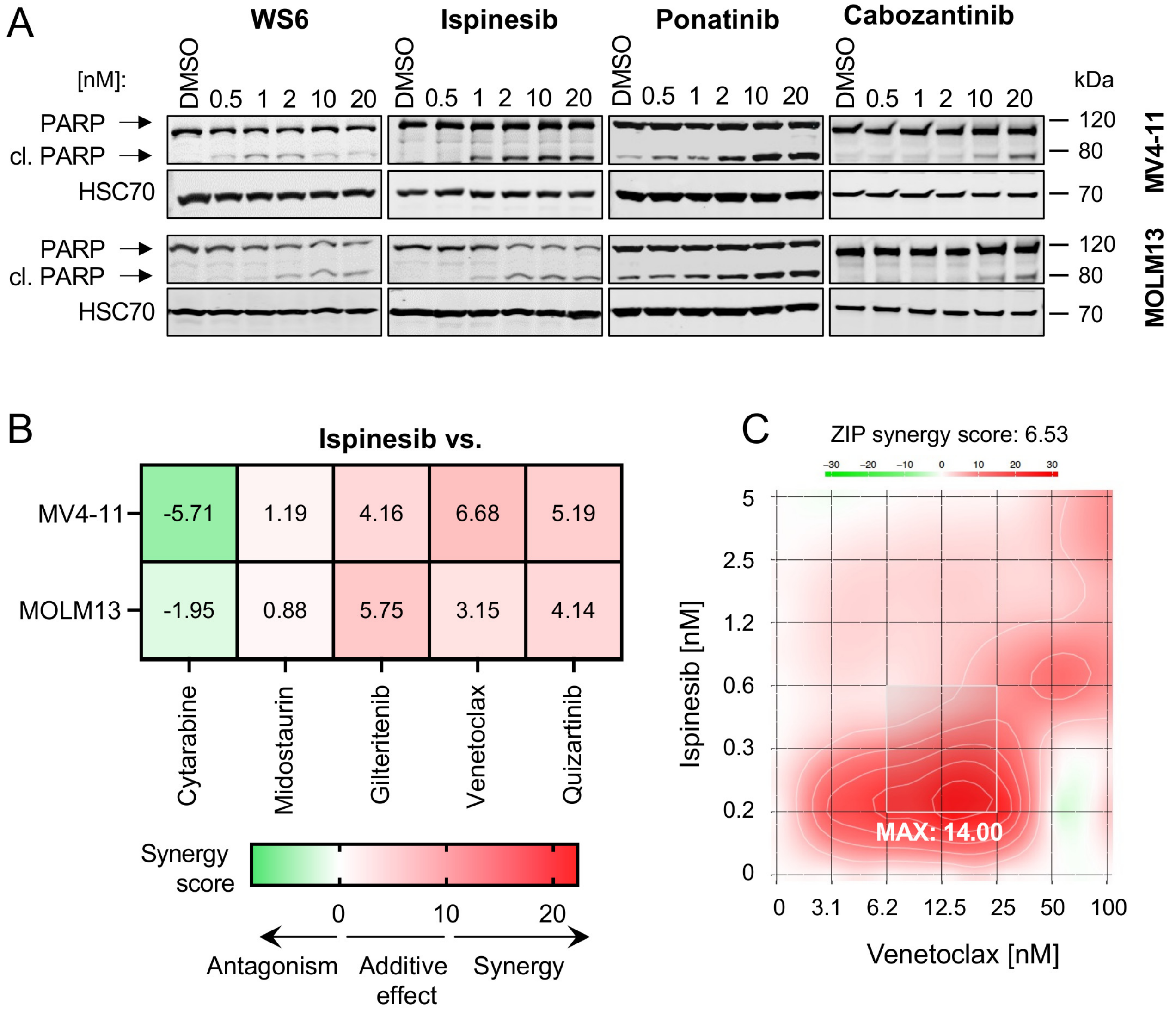

Supplementary Figure S5. Synergy assessment between ispinesib and standard of care drugs. (A) MV4-11 and MOLM13 cells were treated with WS6, ispinesib, cabozantinib or ponatinib at the indicated concentrations for 24 h and immunoblotted against cleaved (cl.) PARP. HSC70 was used as loading control, n=2. Uncropped images can be found in **Figure S8**. **(B)** Heatmap summarizing ZIP scores of the synergy experiments between ispinesib, and standard of care treatments. **(C)** Representative interaction landscape between venetoclax and ispinesib in MV4-11. Most synergistic area is shaded and maximum synergy score is indicated. ZIP stands for Zero interaction potency, n =3.

For Figure 3A and Supp Figure 3A

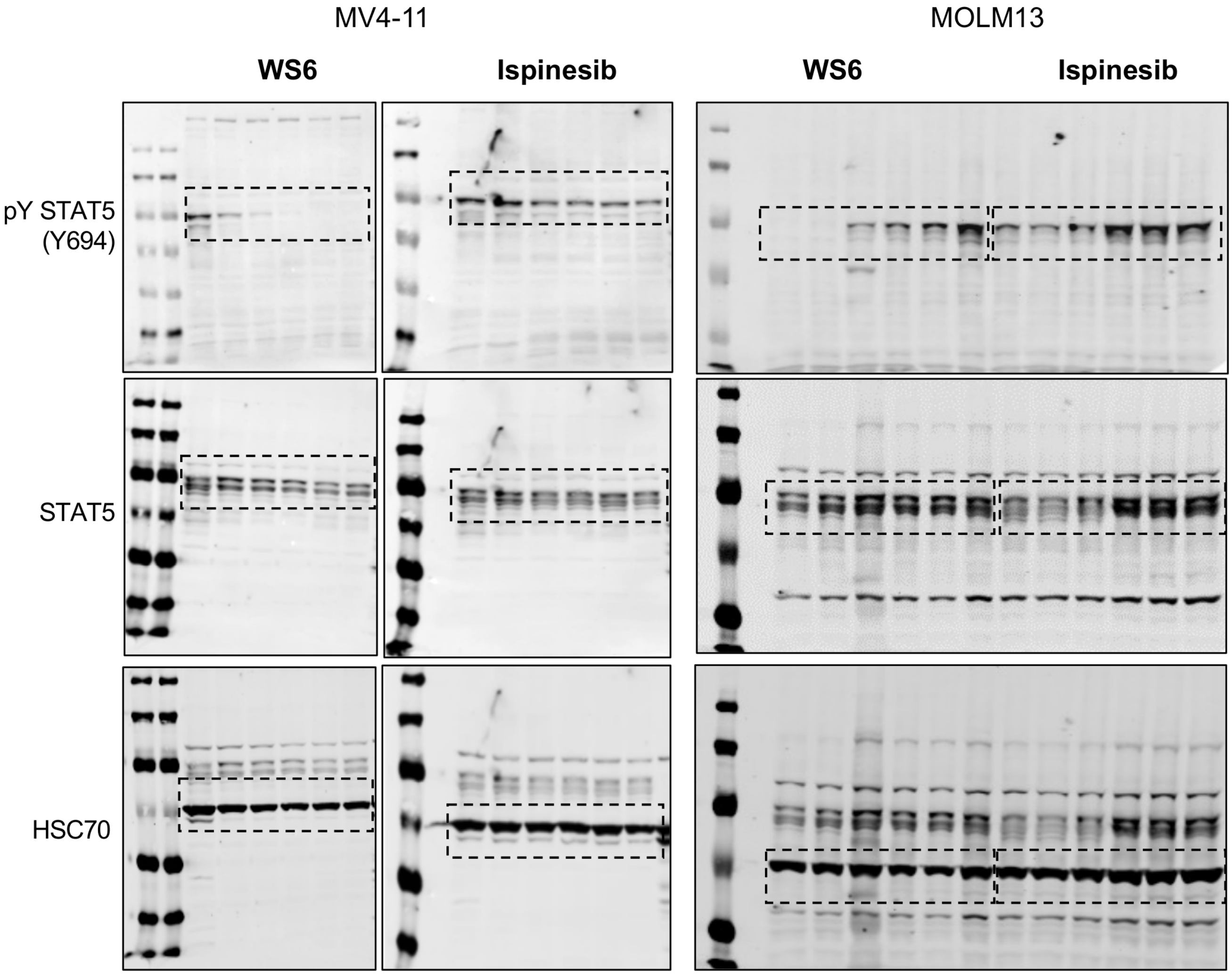

Supplementary Figure S6. Uncropped western blots for Figure 3A and S3A.

For Figure 3A and Supp Figure 3A

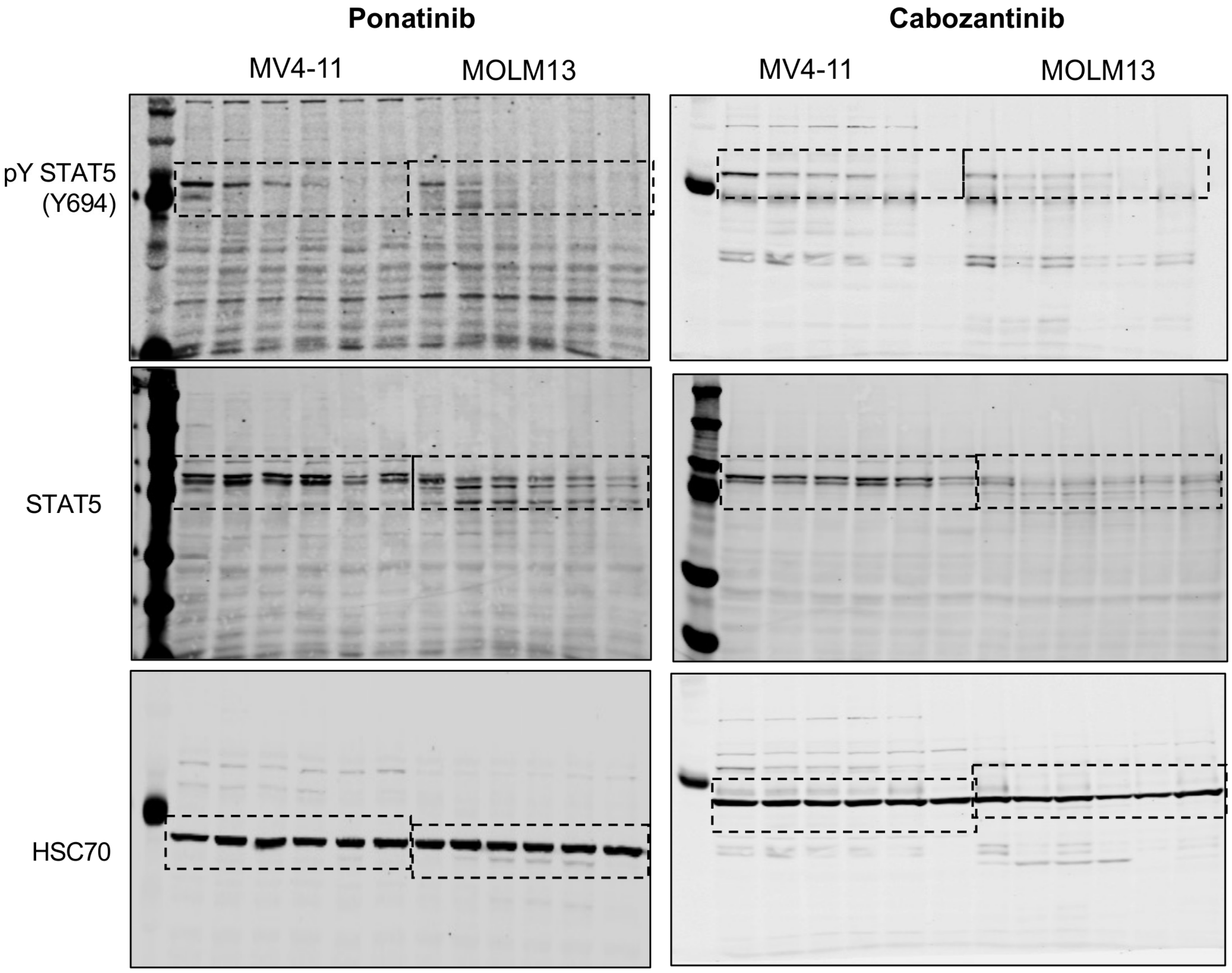

Supplementary Figure S7. Uncropped western blots for Figure 3A and S3A.

For Supp Figure 5A

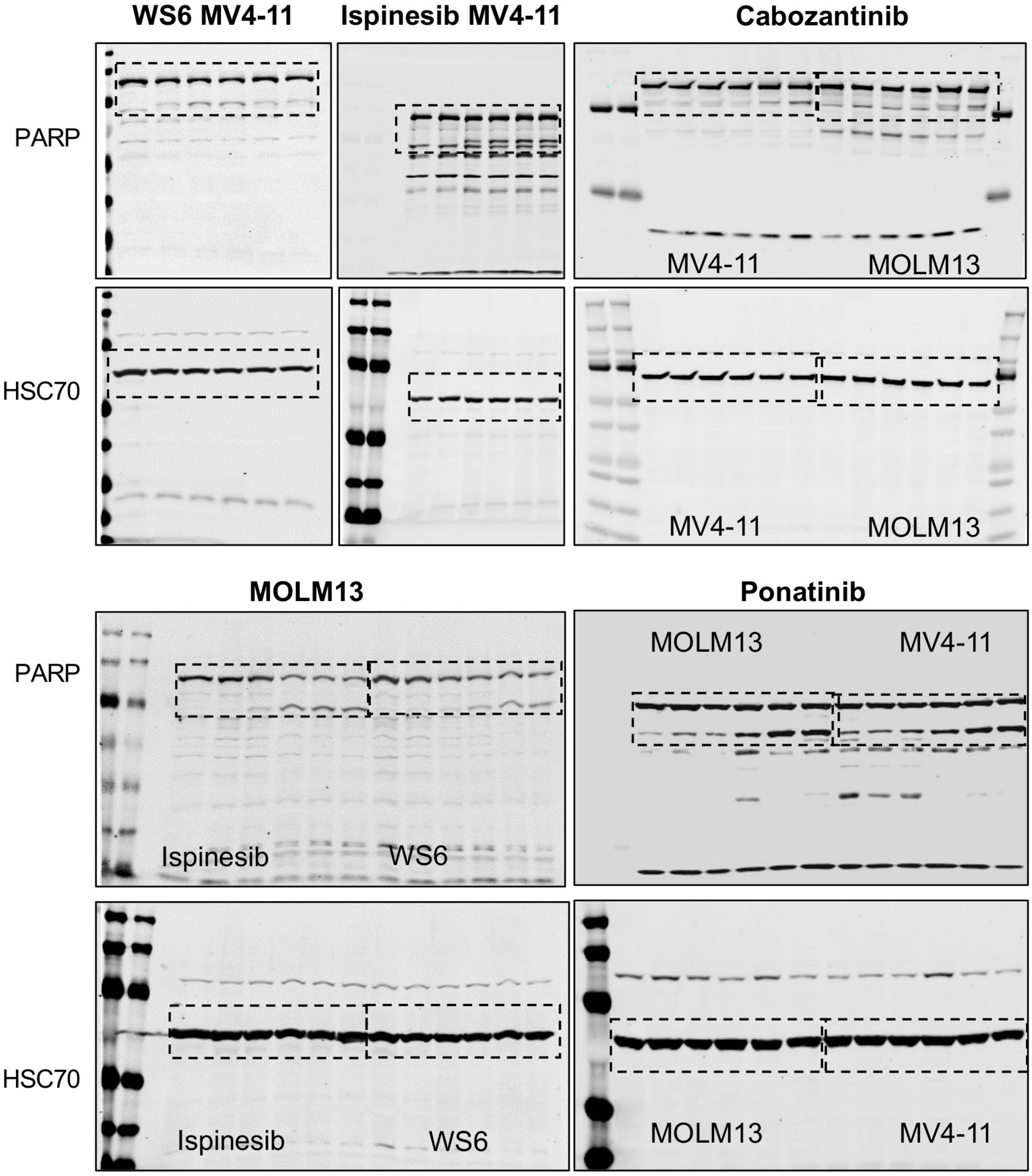

Supplementary Figure S8. Uncropped western blots for Figure S5A
